# Vesicle trafficking and pathways to neurodegeneration

**DOI:** 10.1186/s13024-021-00480-1

**Published:** 2021-08-21

**Authors:** Craig Blackstone, Fiona Elwood, Helene Plun-Favreau, Patrick A. Lewis

**Affiliations:** 1 Department of Neurology, Massachusetts General Hospital and Harvard Medical School, Boston, MA 02114 USA; 2 Novartis Institute for Biomedical Research, 250 Massachusetts Ave, Cambridge, MA 02139 USA; 3 UCL Queen Square Institute of Neurology, Queen Square, London, WC1N 3BG UK; 4 Royal Veterinary College, University of London, London, NW1 0TU UK

Neurodegenerative diseases, encompassing a diverse range of inherited and sporadic disorders characterised by progressive loss of relatively discrete neuronal populations, are a significant and increasing challenge to human health and the global economy [1]. Despite significant advances in our understanding of the underlying ætiology of diseases such as Alzheimer’s, Parkinson’s and Huntington’s, and intense efforts targeting the development of disease-modifying therapies for these disorders, for the majority of people living with neurodegenerative conditions the prognosis remains poor [2–4]. Improving our knowledge of the underlying causes of neuronal loss in these disorders with the goal of developing novel disease-modifying therapies is thus a top priority for research, patient and care-giver communities.

An area of cell biology that has emerged over the past two decades as a key contributor to the events that lead to neuronal cell death across the whole spectrum of neurodegenerative disease is that of vesicle trafficking [5]. Regulating the formation and degradation of vesicles, what goes in to them, where they go, and what happens to them is a fundamental function required for cell viability [6], and so it is perhaps not surprising that dysfunction of these dynamic systems can result in disease. Driven in part by rapid advances in human genetics, it has become very clear that neuronal cells are exquisitely sensitive to disruption of vesicle trafficking – with a wide range of neurodegenerative diseases caused by specific mutations in genes that contribute to the regulation of vesicle trafficking.

To capitalise on the rapid increase in research on vesicle biology in neurodegeneration, a three-day virtual meeting on “Vesicle trafficking and pathways to neurodegeneration” was hosted by Wellcome Connecting Science from May 17th to 19th 2021 (Fig. 1). The goal of this meeting was to bring together researchers from a broad spectrum of neurodegenerative disorders research, including students, early career researchers and established scientists, spanning clinical, genetic, cellular, in vivo, translational biology, and industry in order to break down some of the barriers between these various groups – searching for areas of common interest and opportunities to accelerate the progress of research.

**Fig. 1:**
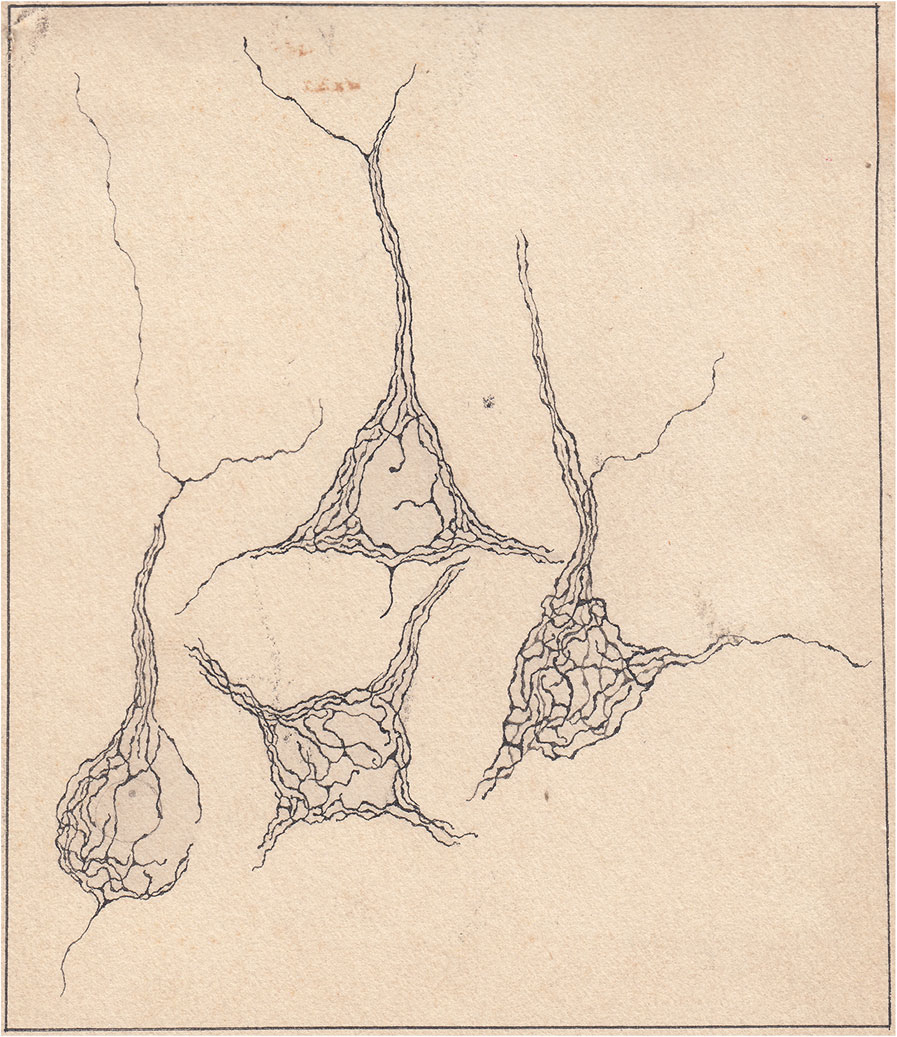
One of the first depictions of intracellular vesicles within the nervous system, drawn by Camillo Golgi, used as the centre piece for the conference proceedings (image courtesy of the University of Pavia)

Like many conferences scheduled over the past 18 months, the original aim was for the meeting to be held in person (in this case at the Wellcome Genome Campus in Hinxton). Circumstances related to the covid-19 pandemic, however, did not allow this and so the meeting was held as a virtual event – with the positive outcome that this opened up attendance throughout the world in a way that would not have been possible in person. The meeting was attended by over 230 researchers, representing 23 different countries, and was divided into sessions covering five broadly-defined areas related to vesicle trafficking (Fig. 2), alongside a session focused on the neurogenetics of vesicle trafficking as well as a drug discovery panel discussing how we can drug vesicle trafficking processes within the brain.

**Fig. 2:**
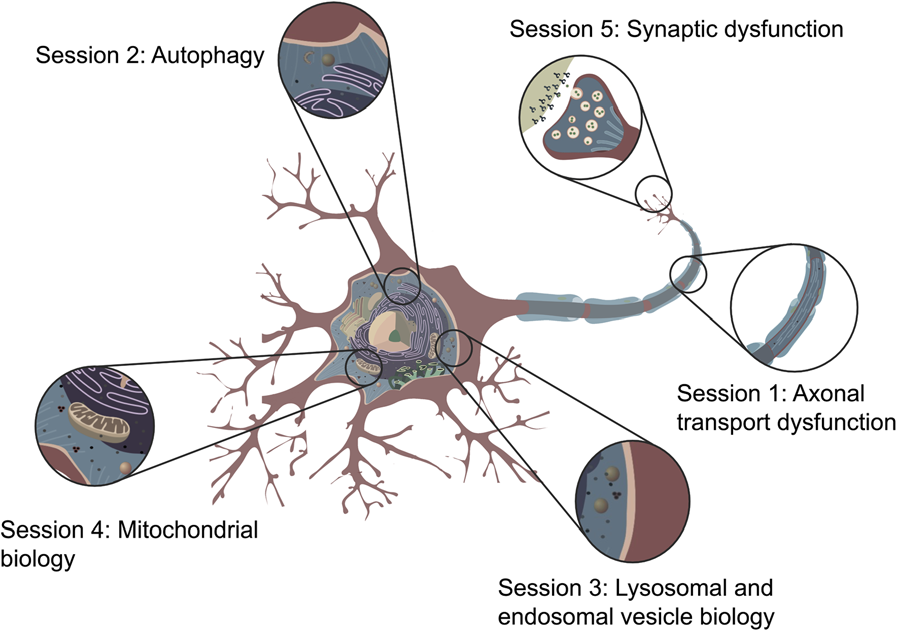
The five main cellular sessions held during the meeting. Image adapted from reference (https://commons.wikimedia.org/wiki/File:Complete_neuron_cell_diagram_en.svg) (image in the public domain)

The conference was bookended by two outstanding keynote lectures, the first from Jennifer Lippincott-Schwartz (Howard Hughes Medical Institute Janelia Research Campus), describing with exquisite resolution the trafficking of proteins from the endoplasmic reticulum through to the Golgi apparatus [7], and the second by Pietro de Camilli (Yale University School of Medicine and Howard Hughes Medical Institute) covering his recent investigations into disruption of vesicle trafficking linked to neurodegenerative disease gene mutations – most notably those linked to *VPS13D* [8].

The intervening sessions showcased some incredibly exciting published and unpublished research, highlighting both the breadth and depth of research into vesicular dysfunction in neurodegeneration. One aspect that became obvious quite quickly was that the somewhat arbitrary dividing lines between different domains of vesicular transport within the cell that was used to demarcate the five biology sessions were just that – somewhat arbitrary. Across all of the sessions one could observe common themes, and indeed common genetic contributors, often acting across multiple different disorders. Making sense of this, and in particular the commonalities and contrasting ætiologies between, for example, vesicular dysfunction contributing to frontal temporal dementia and that found in amyotrophic lateral sclerosis (frequently with closely-related genetic defects) [9], has the potential to reveal important insights into why individuals develop one form of brain disease rather than another.

Two key challenges emerged from the presentations and discussions during the conference. Neither of these are unique to neurodegeneration, but are acutely obvious and – in some respects – perhaps exacerbated by the complexities of studying disorders of the central nervous system. First, the sheer volume of genetic and clinical data that is now being generated across neurological diseases presents a huge task for functional biology. As we develop an ever more detailed understanding of population-wide genetic risk, through large scale sequencing, association studies, and expression analyses, there is an ever longer list of potential risk genes to investigate and comprehend [10]. With regard to this, it was striking that a majority of the presentations at the meeting involved investigating monogenic aspects of neurodegenerative disease, whether that be the ultrastructure of Huntington disease intracellular inclusions and how these disrupt endolysosomal function [11]**,** or the function of Leucine Rich Repeat Kinase 2 in responding to lysosomal damage [12] in Parkinson disease (to cite two examples of topics covered by short talks at the meeting). Moving from a monogenic-centric approach to the cell biology of neurodegeneration to making sense of the complexities of common genetic risk for neurodegeneration at a functional level is a gargantuan task, and one that is only just beginning to be confronted.

The second major challenge is that of translating advances in our understanding of the cellular processes driving disease into clinical benefits for patients. Despite some notable recent successes, for example recent advances in targeting spinal muscular atrophy [13], the development of drugs that modify central nervous system disorders, and in particular neurodegenerative diseases, has proved extremely challenging [14, 15]. Taking dementia as a case study, the last two decades have witnessed a number of promising preclinical drug candidates failing in large human trials [16]. Exploiting the increasing body of knowledge relating to vesicle trafficking dysfunction in neurodegeneration presents some major challenges, not least determining how to achieve specificity in the central nervous system, and how to measure biological activity in a human. The inherent challenges of drugging these pathways may require new approaches in compound screening, model development, the science of therapeutics and biomarker discovery (the subject of some discussion during the panel held as part of this conference). These challenges, however, should not distract from the opportunities presented by the increasing diversity of targets for neurodegeneration and the new insights into disease biology provided by research into this area.

The overriding impression from this conference, taking into account all of the talks and posters presented at the meeting, is a feeling of optimism for the future, in particular with regard to the power of technology to drive insights into the fundamental biology of vesicle trafficking and into understanding disease ætiology.

Across the programme, we were witness to some outstanding examples of the application of high-content screening [17] and cryoelectron tomography [18], providing a high volume of information and close to atomic resolution. Coupled with deep learning approaches applied to increasingly large genetic and biological datasets [19], this heralds a new era in our understanding of the events regulating vesicle trafficking in the cells of the central nervous system. As novel approaches to in silico imaging allow the refinement of experimental models [20], and proteome-wide investigations begin to achieve a level of comprehensiveness comparable to nucleic acid-based genomic analyses [21], this is clearly an important area of biology to follow closely over the coming years.

## Data Availability

Not applicable.
